# Crystal structure of a Co^II^ coordination polymer with a dipyridyl ligand: *catena*-poly[[bis­(nitrato-κ^2^ *O*,*O*′)cobalt(II)]-μ-*N*-(pyridin-2-ylmeth­yl)pyridine-3-amine-κ^3^ *N*,*N*′:*N*′′]

**DOI:** 10.1107/S205698901701475X

**Published:** 2017-10-20

**Authors:** Suk-Hee Moon, Youngjin Kang, Ki-Min Park

**Affiliations:** a Department of Food and Nutrition, Kyungnam College of Information and Technology, Busan 47011, Republic of Korea; b Division of Science Education, Kangwon National University, Chuncheon 24341, Republic of Korea; c Research institute of Natural Science, Gyeongsang National University, Jinju 52828, Republic of Korea

**Keywords:** crystal structure, dipyridyl-type ligand, cobalt(II), zigzag chain, metal-organic framework

## Abstract

The asymmetric unit of the title compound, [Co(NO_3_)_2_ *L*]_*n*_, *L* = *N*-(pyridine-2-ylmeth­yl)pyridine-3-amine (C_11_H_11_N_3_), contains one Co^II^ centre, two nitrate anions and one *L* ligand in which the C_py_—C—N—C_py_ moiety adopts a *trans* conformation with a torsion angle of −173.1 (3) Å. The coordination geometry of the Co^II^ atom is a distorted penta­gonal bipyramid. One amine N atom from the *L* ligand and four O atoms from two η^2^-nitrato ligands form the basal plane and two pyridyl N atoms from two symmetry-related *L* ligands occupy the apical positions [N—Co—N = 171.86 (11)°]. The displacement of the central Co^II^ atom from the basal plane (r.m.s. deviation = 0.085 Å) is 0.1491 (12) Å. Each bidentate nitrate group is bonded asymmetrically to the cobalt atom in an chelating fashion. The Co^II^ ions are linked by the *L* ligands to form a zigzag chain propagating along the *c*-axis direction. Within the zigzag chain, C—H⋯O hydrogen bonds between the ligands and the nitrate anions are observed. Adjacent zigzag chains are connected *via* inter­molecular π–π stacking inter­actions [centroid-to-centroid distance = 3.844 (2) Å] between the pyridine rings together with N/C—H⋯O hydrogen bonds.

## Abstract 2

The Co^II^ atom in the title compound is seven-coordinate, being bound to two pyridine N atoms, one amine N atom and four O atoms to form a distorted penta­gonal–bipyramidal environment. Each dipyridyl-type ligand links the Co^II^ atoms into polymeric zigzag chains, which are connected *via* inter­molecular π–π stacking inter­actions and N/C—H⋯O hydrogen bonds.

## Chemical context   

Over the past few decades, the continuous efforts have been devoted to the design and development of metal–organic frameworks (MOFs) obtained by linking transition metal centers with several organic bridging ligands. In particular, rigid or flexible dipyridyl-type ligands have been widely used to construct MOFs with attractive structures and potential applications in materials chemistry (Silva *et al.*, 2015 ▸; Furukawa *et al.*, 2014 ▸; Wang *et al.*, 2012 ▸; Leong & Vittal, 2011 ▸). Our group has also tried to develop diverse dipyridyl-type MOFs with intriguing topologies including a cyclic dimer (Moon *et al.*, 2011 ▸), zigzag chain (Moon *et al.*, 2016 ▸), double helical chain (Lee *et al.*, 2015 ▸), helical looped-chain (Ju *et al.*, 2014 ▸) and two-dimensional pseudo-polyrotaxane network (Im *et al.*, 2017 ▸), and reported their crystal structures. As a part of our ongoing efforts to develop dipyridyl-type MOFs with different structural motifs, we prepared the title compound obtained by the reaction of cobalt(II) nitrate with a dipyridyl ligand, namely *N*-(pyridine-2-ylmeth­yl)pyridine-3-amine. Herein, we report its crystal structure, which is the first example of a Co^II^ complex with an *N*-(pyridine-2-ylmeth­yl)pyridine-3-amine ligand.
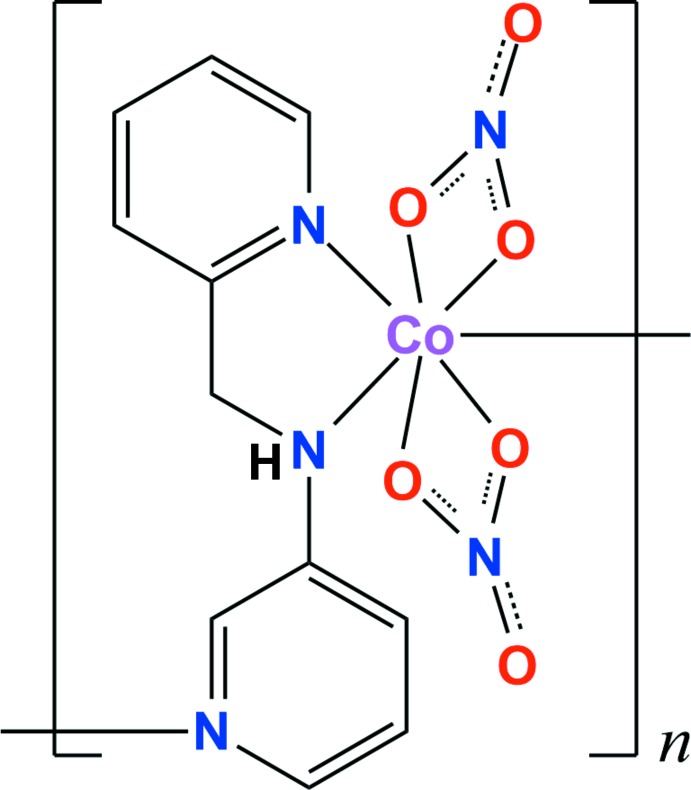


## Structural commentary   

**Figure 1:**
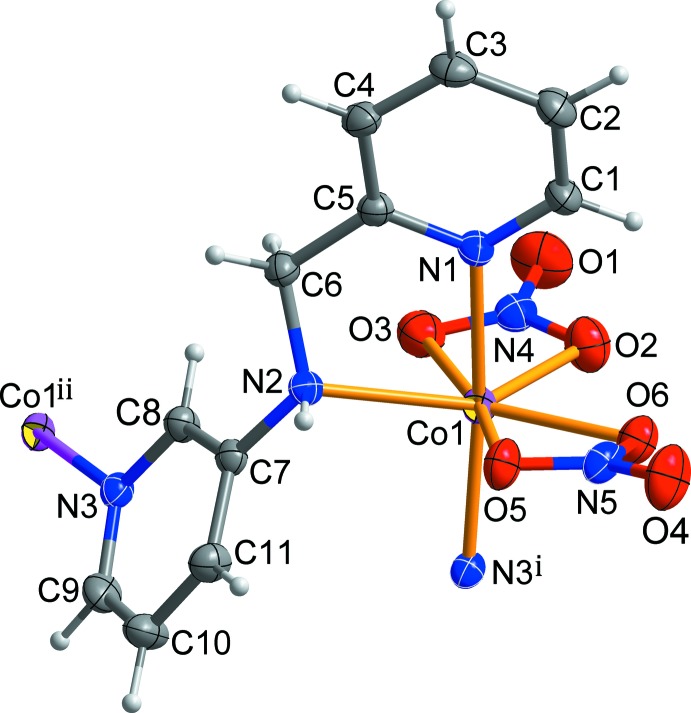
A view of the mol­ecular structure of the title compound, showing the atom-numbering scheme [symmetry codes: (i) *x*, −*y* + 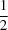, *z* − 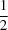; (ii) *x*, −*y* + 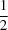, *z* + 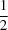]. Displacement ellipsoids are drawn at the 30% probability level. H atoms are shown as small spheres of arbitrary radius.

The asymmetric unit of the title compound comprises one Co^II^ atom, one *L* ligand and two nitrate anions, which coordinate the cobalt ion in a bidentate chelating fashion. The coordin­ation geometry of the Co^II^ atom is distorted penta­gonal bipyramidal with the five basal sites being occupied by one amine N atom from the *L* ligand and four O atoms from two *η* ^2^-nitrato ligands and the two apical positions occupied by two pyridyl N atoms from two symmetry-related *L* ligands [N1—Co1—N3^i^ = 171.86 (11)°; symmetry code: (i) *x*, −*y* + 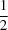, *z* − 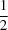] (Fig. 1 ▸). The central Co^II^ atom is displaced by 0.1491 (12) Å from the basal plane (r.m.s. deviation = 0.085 Å). The Co—N distances in apical positions [Co1—N1 = 2.120 (3), Co1—N3^i^ = 2.125 (3) Å] are slightly shorter than that of the basal [Co1—N2 = 2.191 (3) Å]. The largest deviations from the NO_4_ basal plane around the cobalt center involve the angles O2—Co1—O3 [55.81 (11)°] and N2—Co1—O5 [84.19 (9)°]. This distortion may reflect the narrow bite angles of the bidentate nitrate ions.

**Figure 2:**
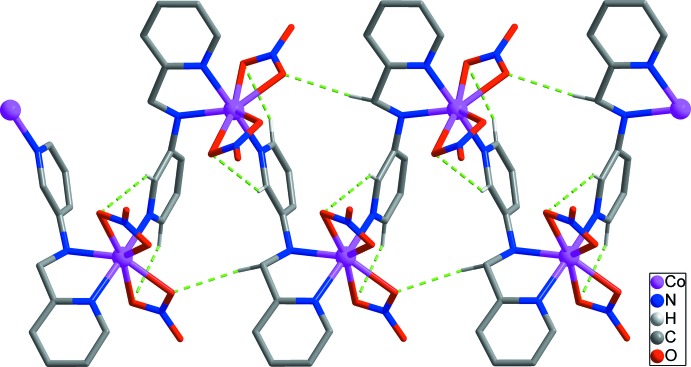
The zigzag chain formed through C—H⋯O hydrogen bonds (green dashed lines). H atoms not involved in inter­molecular inter­actions have been omitted for clarity.

The *L* ligand adopts a stretched *trans* conformation with the C5—C6—N2—C7 torsion angle being −173.1 (3) Å. The terminal pyridine rings of the *L* ligand are nearly perpendic­ular to each other, with the dihedral angle between their mean planes being 76.74 (12)°. Each bidentate nitrate group is bonded asymmetrically to the cobalt atom [Co1—O2 = 2.139 (3), Co1—O3 = 2.327 (3), Co1—O5 = 2.365 (2) and Co1—O6 = 2.167 (2) Å]. Each *L* ligand is bridged by the Co^II^ ions, forming –(Co-*L*)_*n*_– zigzag chains propagating along the *c*-axis direction (Figs. 2 ▸ and 3 ▸). The zigzag chain is reinforced by several C—H⋯O hydrogen bonds (Table 2 ▸; green dashed lines in Fig. 2 ▸) between the *L* ligands and the nitrate O atoms.

## Supra­molecular features   

**Figure 3:**
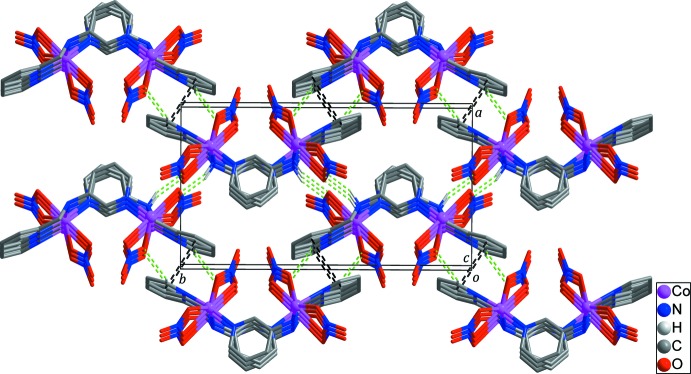
The three-dimensional structure formed through inter­molecular π–π stacking inter­actions (black dashed lines) and N/C—H⋯O hydrogen bonds (green dashed lines). H atoms not involved in inter­molecular inter­actions have been omitted for clarity.

In the crystal of the title compound, adjacent zigzag chains are linked by inter­molecular π–π stacking inter­actions [black dashed lines in Fig. 3 ▸; *Cg*1⋯*Cg*1^ii^ = 3.844 (2) Å; *Cg*1 is the centroid of the N1/C1–C5 ring; symmetry code: (ii) −*x*, −*y* + 1, −*z* + 1] between the pyridine rings and C—H⋯O hydrogen bonds between pyridyl H atoms and nitrate O atoms (Table 1 ▸; green dashed lines in Fig. 3 ▸), forming layers extending parallel to the (100) plane. The layers are further connected by inter­molecular N—H⋯O hydrogen bonds (Table 2 ▸; green dashed lines in Fig. 3 ▸) between amine H atoms and nitrate O atoms.

## Database survey   

A search of the Cambridge Structural Database (Version 5.38, update May 2017; Groom *et al.*, 2016 ▸) for the compounds obtained by the reaction of transition metal ions and the *L* ligand gave 11 hits. Three (AQEGAG, AQEGEK, AQEGIO) are Hg^II^ complexes and seven (CEZPAA, DURFON, POFKUS, PONTUJ, VIPTOF, WIHWUH, WIHXOC) of them are Ag^I^ complexes. The remaining one is a Zn^II^ complex (DUVPER). There are no metal complexes that are similar to the structure of the Co^II^ complex described above. Therefore, the title compound is the first example of a Co^II^ complex with an *L* ligand.

## Synthesis and crystallization   

The *L* ligand was synthesized according to a literature method (Lee *et al.*, 2013 ▸). X-ray-quality single crystals of the title compound were obtained by slow evaporation of an aceto­nitrile solution of the *L* ligand with Co(NO_3_)_2_·6H_2_O in the molar ratio 1:1.

## Refinement   

Crystal data, data collection and structure refinement details are summarized in Table 3 ▸. The amine H atom was located from a difference-Fourier map and refined with riding constraints [*d*(N—H) = 0.96 Å]. All other H atoms were positioned geometrically and refined as riding, with *d*(C—H) = 0.93 Å for C*sp* ^2^—H and 0.97 Å for methyl­ene C—H. For all H atoms, *U*_iso_(H) = 1.2*U*_eq_ of the parent atom.

## Supplementary Material

Crystal structure: contains datablock(s) I, New_Global_Publ_Block. DOI: 10.1107/S205698901701475X/nk2241sup1.cif

Structure factors: contains datablock(s) I. DOI: 10.1107/S205698901701475X/nk2241Isup2.hkl

CCDC reference: 1579473

Additional supporting information:  crystallographic information; 3D view; checkCIF report

## Figures and Tables

**Table 1:** Selected geometric parameters (Å, °)

Co1—N1	2.120 (3)	Co1—N2	2.191 (3)
Co1—N3^i^	2.125 (3)	Co1—O3	2.327 (3)
Co1—O2	2.139 (3)	Co1—O5	2.365 (2)
Co1—O6	2.167 (2)		
			
N1—Co1—N3^i^	171.86 (11)	N3^i^—Co1—O3	92.35 (10)
N1—Co1—O2	90.67 (11)	O2—Co1—O3	55.81 (11)
N3^i^—Co1—O2	97.46 (11)	O6—Co1—O3	135.30 (10)
N1—Co1—O6	90.57 (10)	N2—Co1—O3	84.11 (10)
N3^i^—Co1—O6	90.67 (10)	N1—Co1—O5	81.99 (10)
O2—Co1—O6	79.58 (11)	N3^i^—Co1—O5	92.07 (10)
N1—Co1—N2	77.43 (10)	O2—Co1—O5	134.56 (10)
N3^i^—Co1—N2	96.52 (10)	O6—Co1—O5	55.89 (9)
O2—Co1—N2	137.86 (11)	N2—Co1—O5	84.19 (9)
O6—Co1—N2	139.71 (10)	O3—Co1—O5	167.89 (10)
N1—Co1—O3	92.42 (10)		

Symmetry code: (i) 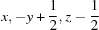.

**Table 2:** Hydrogen-bond geometry (Å, °)

*D*—H⋯*A*	*D*—H	H⋯*A*	*D*⋯*A*	*D*—H⋯*A*
N2—H2*N*⋯O5^ii^	0.96	2.11	2.987 (4)	151
C1—H1⋯O2^iii^	0.93	2.57	3.186 (5)	124
C6—H6*A*⋯O6^iv^	0.97	2.54	3.413 (4)	149
C8—H8⋯O3^v^	0.93	2.53	3.163 (4)	126
C9—H9⋯O5^v^	0.93	2.49	3.164 (4)	130

Symmetry codes: (ii) 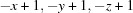; (iii) 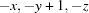; (iv) 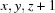; (v) 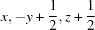.

**Table 3:** Experimental details

Crystal data	
Chemical formula	[Co(NO_3_)_2_(C_11_H_11_N_3_)]
*M*_r_	368.18
Crystal system, space group	Monoclinic, *P*2_1_/*c*
Temperature (K)	298
*a*, *b*, *c* (Å)	10.4550 (13), 17.662 (2), 7.9653 (10)
β (°)	108.160 (3)
*V* (Å^3^)	1397.6 (3)
*Z*	4
Radiation type	Mo *K*α
μ (mm^−1^)	1.27
Crystal size (mm)	0.32 × 0.27 × 0.23

Data collection	
Diffractometer	Bruker APEXII CCD
Absorption correction	Multi-scan (*SADABS*; Bruker, 2014 ▸)
*T*_min_, *T*_max_	0.644, 0.725
No. of measured, independent and observed [*I* > 2σ(*I*)] reflections	7841, 2737, 1695
*R*_int_	0.070
(sin θ/λ)_max_ (Å^−1^)	0.617

Refinement	
*R*[*F* ^2^ > 2σ(*F* ^2^)], *wR*(*F* ^2^), *S*	0.041, 0.087, 0.96
No. of reflections	2737
No. of parameters	208
H-atom treatment	H-atom parameters constrained
Δρ_max_, Δρ_min_ (e Å^−3^)	0.26, −0.31

Computer programs: *APEX2* and *SAINT* (Bruker, 2014 ▸), *SHELXS97* (Sheldrick, 2008 ▸), *SHELXL2014* (Sheldrick, 2015 ▸), *DIAMOND* (Brandenburg, 2010 ▸), *SHELXTL* (Sheldrick, 2008 ▸) and *publCIF* (Westrip, 2010 ▸).

## supplementary crystallographic information

### Crystal data

**Table:**

[Co(NO_3_)_2_(C_11_H_11_N_3_)]	*F*(000) = 748
*M**_r_* = 368.18	*D*_x_ = 1.750 Mg m^−^^3^
Monoclinic, *P*2_1_/*c*	Mo *K*α radiation, λ = 0.71073 Å
*a* = 10.4550 (13) Å	Cell parameters from 2739 reflections
*b* = 17.662 (2) Å	θ = 2.1–26.0°
*c* = 7.9653 (10) Å	µ = 1.27 mm^−^^1^
β = 108.160 (3)°	*T* = 298 K
*V* = 1397.6 (3) Å^3^	Block, violet
*Z* = 4	0.32 × 0.27 × 0.23 mm

### Data collection

**Table:**

Bruker APEXII CCD diffractometer	1695 reflections with *I* > 2σ(*I*)
φ and ω scans	*R*_int_ = 0.070
Absorption correction: multi-scan (SADABS; Bruker, 2014)	θ_max_ = 26.0°, θ_min_ = 2.4°
*T*_min_ = 0.644, *T*_max_ = 0.725	*h* = −8→12
7841 measured reflections	*k* = −21→18
2737 independent reflections	*l* = −9→9

### Refinement

**Table:**

Refinement on *F*^2^	0 restraints
Least-squares matrix: full	Hydrogen site location: mixed
*R*[*F*^2^ > 2σ(*F*^2^)] = 0.041	H-atom parameters constrained
*wR*(*F*^2^) = 0.087	*w* = 1/[σ^2^(*F*_o_^2^) + (0.0331*P*)^2^] where *P* = (*F*_o_^2^ + 2*F*_c_^2^)/3
*S* = 0.96	(Δ/σ)_max_ < 0.001
2737 reflections	Δρ_max_ = 0.26 e Å^−^^3^
208 parameters	Δρ_min_ = −0.31 e Å^−^^3^

### Special details

**Table:**

Geometry. All esds (except the esd in the dihedral angle between two l.s. planes) are estimated using the full covariance matrix. The cell esds are taken into account individually in the estimation of esds in distances, angles and torsion angles; correlations between esds in cell parameters are only used when they are defined by crystal symmetry. An approximate (isotropic) treatment of cell esds is used for estimating esds involving l.s. planes.

### Fractional atomic coordinates and isotropic or equivalent isotropic displacement parameters (Å^2^)

**Table:**

	*x*	*y*	*z*	*U*_iso_*/*U*_eq_	
Co1	0.27153 (4)	0.39860 (2)	0.26458 (6)	0.03391 (16)	
N1	0.1788 (3)	0.48757 (15)	0.3629 (3)	0.0358 (7)	
N2	0.3601 (3)	0.38625 (14)	0.5512 (3)	0.0326 (7)	
H2N	0.4398	0.4164	0.5764	0.039*	
N3	0.3901 (3)	0.18597 (14)	0.6941 (3)	0.0343 (7)	
C1	0.1212 (4)	0.54840 (19)	0.2707 (5)	0.0431 (9)	
H1	0.1017	0.5474	0.1486	0.052*	
C2	0.0893 (4)	0.6122 (2)	0.3468 (5)	0.0471 (10)	
H2	0.0516	0.6541	0.2786	0.057*	
C3	0.1144 (4)	0.6125 (2)	0.5272 (5)	0.0471 (10)	
H3	0.0940	0.6550	0.5829	0.056*	
C4	0.1700 (3)	0.5495 (2)	0.6242 (5)	0.0398 (9)	
H4	0.1869	0.5488	0.7459	0.048*	
C5	0.2005 (3)	0.48701 (18)	0.5379 (4)	0.0330 (8)	
C6	0.2606 (4)	0.41626 (18)	0.6310 (4)	0.0383 (9)	
H6A	0.3042	0.4268	0.7552	0.046*	
H6B	0.1905	0.3790	0.6213	0.046*	
C7	0.4171 (3)	0.31400 (18)	0.6103 (4)	0.0303 (8)	
C8	0.3417 (3)	0.25586 (18)	0.6498 (4)	0.0345 (8)	
H8	0.2533	0.2660	0.6453	0.041*	
C9	0.5162 (4)	0.1726 (2)	0.6967 (5)	0.0453 (9)	
H9	0.5501	0.1238	0.7224	0.054*	
C10	0.5989 (4)	0.2273 (2)	0.6632 (5)	0.0489 (10)	
H10	0.6870	0.2160	0.6689	0.059*	
C11	0.5489 (4)	0.2985 (2)	0.6216 (5)	0.0427 (9)	
H11	0.6035	0.3366	0.6008	0.051*	
N4	0.0418 (3)	0.32781 (17)	0.1539 (4)	0.0423 (8)	
O1	−0.0745 (3)	0.30287 (19)	0.0943 (4)	0.0832 (10)	
O2	0.0852 (3)	0.37335 (16)	0.0660 (4)	0.0661 (8)	
O3	0.1166 (3)	0.30993 (15)	0.2978 (4)	0.0628 (8)	
N5	0.3877 (3)	0.50903 (17)	0.1413 (4)	0.0412 (8)	
O4	0.4248 (3)	0.56412 (17)	0.0738 (4)	0.0752 (9)	
O5	0.4407 (3)	0.49125 (14)	0.2962 (3)	0.0500 (7)	
O6	0.2877 (3)	0.47059 (14)	0.0518 (3)	0.0517 (7)	

### Atomic displacement parameters (Å^2^)

**Table:**

	*U*^11^	*U*^22^	*U*^33^	*U*^12^	*U*^13^	*U*^23^
Co1	0.0436 (3)	0.0265 (2)	0.0319 (3)	0.0006 (2)	0.0121 (2)	−0.0005 (2)
N1	0.0423 (18)	0.0294 (16)	0.0357 (18)	0.0051 (13)	0.0120 (14)	−0.0007 (14)
N2	0.0346 (16)	0.0276 (16)	0.0370 (16)	0.0011 (12)	0.0132 (13)	0.0031 (13)
N3	0.044 (2)	0.0293 (16)	0.0318 (17)	0.0018 (13)	0.0147 (14)	0.0016 (13)
C1	0.052 (2)	0.039 (2)	0.040 (2)	0.0139 (17)	0.0157 (18)	0.0057 (18)
C2	0.054 (2)	0.033 (2)	0.053 (3)	0.0097 (17)	0.015 (2)	0.0066 (18)
C3	0.049 (2)	0.037 (2)	0.056 (3)	0.0076 (17)	0.019 (2)	−0.0097 (19)
C4	0.042 (2)	0.042 (2)	0.037 (2)	0.0042 (16)	0.0146 (18)	−0.0065 (17)
C5	0.034 (2)	0.0311 (19)	0.036 (2)	0.0020 (14)	0.0133 (16)	0.0005 (16)
C6	0.047 (2)	0.035 (2)	0.035 (2)	0.0059 (16)	0.0164 (17)	0.0011 (16)
C7	0.034 (2)	0.0294 (19)	0.0264 (18)	−0.0007 (15)	0.0080 (15)	−0.0005 (15)
C8	0.034 (2)	0.032 (2)	0.040 (2)	0.0017 (15)	0.0142 (17)	0.0049 (16)
C9	0.058 (3)	0.031 (2)	0.049 (2)	0.0120 (18)	0.020 (2)	0.0066 (17)
C10	0.041 (2)	0.043 (2)	0.070 (3)	0.0103 (18)	0.028 (2)	0.013 (2)
C11	0.044 (3)	0.036 (2)	0.053 (2)	−0.0016 (17)	0.0217 (19)	0.0038 (18)
N4	0.047 (2)	0.0347 (19)	0.047 (2)	−0.0007 (15)	0.0167 (18)	−0.0056 (16)
O1	0.057 (2)	0.088 (3)	0.096 (3)	−0.0211 (18)	0.0120 (18)	−0.014 (2)
O2	0.065 (2)	0.067 (2)	0.0587 (19)	−0.0084 (15)	0.0074 (15)	0.0029 (16)
O3	0.064 (2)	0.061 (2)	0.061 (2)	0.0070 (15)	0.0166 (17)	−0.0011 (16)
N5	0.057 (2)	0.0290 (18)	0.043 (2)	−0.0088 (15)	0.0227 (17)	−0.0023 (15)
O4	0.102 (3)	0.059 (2)	0.066 (2)	−0.0244 (17)	0.0285 (18)	0.0130 (16)
O5	0.0670 (19)	0.0443 (16)	0.0382 (16)	−0.0091 (12)	0.0155 (14)	0.0042 (13)
O6	0.0631 (19)	0.0502 (17)	0.0423 (16)	−0.0101 (14)	0.0173 (14)	−0.0049 (13)

### Geometric parameters (Å, º)

**Table:**

Co1—N1	2.120 (3)	C3—H3	0.9300
Co1—N3^i^	2.125 (3)	C4—C5	1.389 (4)
Co1—O2	2.139 (3)	C4—H4	0.9300
Co1—O6	2.167 (2)	C5—C6	1.488 (4)
Co1—N2	2.191 (3)	C6—H6A	0.9700
Co1—O3	2.327 (3)	C6—H6B	0.9700
Co1—O5	2.365 (2)	C7—C11	1.381 (4)
N1—C1	1.334 (4)	C7—C8	1.389 (4)
N1—C5	1.341 (4)	C8—H8	0.9300
N2—C7	1.425 (4)	C9—C10	1.378 (5)
N2—C6	1.475 (4)	C9—H9	0.9300
N2—H2N	0.9565	C10—C11	1.362 (5)
N3—C9	1.334 (4)	C10—H10	0.9300
N3—C8	1.339 (4)	C11—H11	0.9300
N3—Co1^ii^	2.125 (3)	N4—O3	1.211 (4)
C1—C2	1.368 (4)	N4—O1	1.240 (4)
C1—H1	0.9300	N4—O2	1.241 (4)
C2—C3	1.378 (5)	N5—O5	1.226 (3)
C2—H2	0.9300	N5—O4	1.232 (3)
C3—C4	1.376 (5)	N5—O6	1.263 (4)
			
N1—Co1—N3^i^	171.86 (11)	C4—C3—H3	120.2
N1—Co1—O2	90.67 (11)	C2—C3—H3	120.2
N3^i^—Co1—O2	97.46 (11)	C3—C4—C5	119.1 (3)
N1—Co1—O6	90.57 (10)	C3—C4—H4	120.4
N3^i^—Co1—O6	90.67 (10)	C5—C4—H4	120.4
O2—Co1—O6	79.58 (11)	N1—C5—C4	121.2 (3)
N1—Co1—N2	77.43 (10)	N1—C5—C6	115.6 (3)
N3^i^—Co1—N2	96.52 (10)	C4—C5—C6	123.2 (3)
O2—Co1—N2	137.86 (11)	N2—C6—C5	109.4 (3)
O6—Co1—N2	139.71 (10)	N2—C6—H6A	109.8
N1—Co1—O3	92.42 (10)	C5—C6—H6A	109.8
N3^i^—Co1—O3	92.35 (10)	N2—C6—H6B	109.8
O2—Co1—O3	55.81 (11)	C5—C6—H6B	109.8
O6—Co1—O3	135.30 (10)	H6A—C6—H6B	108.2
N2—Co1—O3	84.11 (10)	C11—C7—C8	117.7 (3)
N1—Co1—O5	81.99 (10)	C11—C7—N2	120.4 (3)
N3^i^—Co1—O5	92.07 (10)	C8—C7—N2	121.9 (3)
O2—Co1—O5	134.56 (10)	N3—C8—C7	123.1 (3)
O6—Co1—O5	55.89 (9)	N3—C8—H8	118.4
N2—Co1—O5	84.19 (9)	C7—C8—H8	118.4
O3—Co1—O5	167.89 (10)	N3—C9—C10	123.3 (3)
C1—N1—C5	118.6 (3)	N3—C9—H9	118.3
C1—N1—Co1	125.0 (2)	C10—C9—H9	118.3
C5—N1—Co1	115.4 (2)	C11—C10—C9	118.6 (3)
C7—N2—C6	117.2 (2)	C11—C10—H10	120.7
C7—N2—Co1	115.52 (19)	C9—C10—H10	120.7
C6—N2—Co1	106.71 (19)	C10—C11—C7	119.9 (3)
C7—N2—H2N	100.5	C10—C11—H11	120.0
C6—N2—H2N	113.2	C7—C11—H11	120.0
Co1—N2—H2N	102.7	O3—N4—O1	122.4 (3)
C9—N3—C8	117.3 (3)	O3—N4—O2	117.5 (3)
C9—N3—Co1^ii^	121.8 (2)	O1—N4—O2	120.1 (4)
C8—N3—Co1^ii^	120.9 (2)	N4—O2—Co1	97.3 (2)
N1—C1—C2	123.4 (3)	N4—O3—Co1	89.1 (2)
N1—C1—H1	118.3	O5—N5—O4	122.4 (3)
C2—C1—H1	118.3	O5—N5—O6	117.7 (3)
C1—C2—C3	118.1 (3)	O4—N5—O6	119.7 (3)
C1—C2—H2	120.9	N5—O5—Co1	88.92 (19)
C3—C2—H2	120.9	N5—O6—Co1	97.3 (2)
C4—C3—C2	119.5 (3)		
			
C5—N1—C1—C2	3.5 (5)	C9—N3—C8—C7	−1.1 (5)
Co1—N1—C1—C2	−165.0 (3)	Co1^ii^—N3—C8—C7	177.1 (2)
N1—C1—C2—C3	−2.0 (5)	C11—C7—C8—N3	−1.4 (5)
C1—C2—C3—C4	−0.1 (5)	N2—C7—C8—N3	175.4 (3)
C2—C3—C4—C5	0.5 (5)	C8—N3—C9—C10	2.6 (5)
C1—N1—C5—C4	−3.0 (5)	Co1^ii^—N3—C9—C10	−175.6 (3)
Co1—N1—C5—C4	166.5 (2)	N3—C9—C10—C11	−1.5 (6)
C1—N1—C5—C6	177.9 (3)	C9—C10—C11—C7	−1.3 (5)
Co1—N1—C5—C6	−12.5 (4)	C8—C7—C11—C10	2.6 (5)
C3—C4—C5—N1	1.1 (5)	N2—C7—C11—C10	−174.2 (3)
C3—C4—C5—C6	−179.9 (3)	O3—N4—O2—Co1	−6.4 (3)
C7—N2—C6—C5	−173.1 (3)	O1—N4—O2—Co1	173.0 (3)
Co1—N2—C6—C5	−41.8 (3)	O1—N4—O3—Co1	−173.5 (3)
N1—C5—C6—N2	37.5 (4)	O2—N4—O3—Co1	5.8 (3)
C4—C5—C6—N2	−141.5 (3)	O4—N5—O5—Co1	173.0 (3)
C6—N2—C7—C11	−145.9 (3)	O6—N5—O5—Co1	−4.0 (3)
Co1—N2—C7—C11	87.0 (3)	O5—N5—O6—Co1	4.4 (3)
C6—N2—C7—C8	37.4 (4)	O4—N5—O6—Co1	−172.7 (3)
Co1—N2—C7—C8	−89.8 (3)		

Symmetry codes: (i) *x*, −*y*+1/2, *z*−1/2; (ii) *x*, −*y*+1/2, *z*+1/2.

### Hydrogen-bond geometry (Å, º)

**Table:**

*D*—H···*A*	*D*—H	H···*A*	*D*···*A*	*D*—H···*A*
N2—H2*N*···O5^iii^	0.96	2.11	2.987 (4)	151
C1—H1···O2^iv^	0.93	2.57	3.186 (5)	124
C6—H6*A*···O6^v^	0.97	2.54	3.413 (4)	149
C8—H8···O3^ii^	0.93	2.53	3.163 (4)	126
C9—H9···O5^ii^	0.93	2.49	3.164 (4)	130

Symmetry codes: (ii) *x*, −*y*+1/2, *z*+1/2; (iii) −*x*+1, −*y*+1, −*z*+1; (iv) −*x*, −*y*+1, −*z*; (v) *x*, *y*, *z*+1.
